# Myelination of Purkinje axons is critical for resilient synaptic transmission in the deep cerebellar nucleus

**DOI:** 10.1038/s41598-018-19314-0

**Published:** 2018-01-18

**Authors:** Tara Barron, Julia Saifetiarova, Manzoor A. Bhat, Jun Hee Kim

**Affiliations:** Department of Cellular and Integrative Physiology, University of Texas Health Science Center, San Antonio, Texas 78229 USA

## Abstract

The roles of myelin in maintaining axonal integrity and action potential (AP) propagation are well established, but its role in synapse maintenance and neurotransmission remains largely understudied. Here, we investigated how Purkinje axon myelination regulates synaptic transmission in the Purkinje to deep cerebellar nuclei (DCN) synapses using the *Long Evans Shaker* (*LES*) rat, which lacks compact myelin and thus displays severe locomotion deficits. DCN neurons fired spontaneous action potentials (APs), whose frequencies were dependent on the extent of myelin. In the *LES* cerebellum with severe myelin deficiency, DCN neurons were hyper-excitable, exhibiting spontaneous AP firing at a much higher frequency compared to those from wild type (*LE*) and heterozygote (*LEHet*) rats. The hyper-excitability in *LES* DCN neurons resulted from reduced inhibitory GABAergic inputs from Purkinje cells to DCN neurons. Corresponding with functional alterations including failures of AP propagation, electron microscopic analysis revealed anatomically fewer active zones at the presynaptic terminals of Purkinje cells in both *LEHet* and *LES* rats. Taken together, these studies suggest that proper axonal myelination critically regulates presynaptic terminal structure and function and directly impacts synaptic transmission in the Purkinje cell-DCN cell synapse in the cerebellum.

## Introduction

Myelin sheaths wrap around axons to create electrical insulation, increasing efficiency of AP conduction and maintenance and protection of axons from damage due to injury or disease^1–3^. Defects in central myelination can lead to neurodegenerative diseases, such as multiple sclerosis (MS)^2,4,5^. When the myelination process is impaired, axons are unable to carry APs at their highest efficiency, leading to slower conduction velocity or failures of AP propagation^6^ that can be rescued with remyelination^7^. In addition to an increase in the central conduction time, dys-/demyelination alters synaptic neurotransmission in the auditory nervous system^8^. The reduction in the temporal fidelity of AP firing and synaptic transmission caused by dys-/demyelination critically influences the synchrony of neuronal activities with sub-millisecond accuracy in the neurosensory and motor system^6,8,9^.

Central demyelination is the primary cause of symptoms in MS, including tremor and lack of motor coordination. It has been recently suggested that AP firing of Purkinje cells in the cerebellum is highly synchronous^10–12^ and that this temporal synchrony of APs is important for motor coordination^12^. In dys-/demyelination, the temporal synchrony of conduction along the highly myelinated Purkinje axon could be easily disrupted by conduction inefficiency due to myelin loss, and consequently affect the synaptic outputs of these axons to the DCN. DCN neurons have been characterized previously and have been shown to exhibit spontaneous AP firing^13,14^. The excitability of DCN neurons is regulated by GABA-mediated inhibitory inputs from Purkinje cells and glutamate-mediated excitable inputs from climbing and mossy fibers. In addition to axonal deficits, synaptic dysfunction and loss also plays a critical role in cerebellar pathology during progressive MS^15,16^. A recent study in the post-mortem cerebellum of an MS patient demonstrated a reduction in the number and density of axosomatic synapses, as well as widened intercellular clefts between pre-and post-synaptic sites in the DCN^15^. Glutamatergic neurotransmission alterations independent of myelination have been implicated in experimental autoimmune encephalomyelitis, a rodent model of MS^17^. However, the impact of demyelination without immune insult on synaptic transmission is still unknown. In particular, structural and functional consequences in GABAergic Purkinje terminal-DCN neuron synapses remain largely unexplored, although the DCN is a prominent site for lesion development in demyelinating conditions.

The present study demonstrates how lack of myelination impacts synaptic function in the cerebellum using the *LES* rat, which has a genetic deletion of myelin basic protein (MBP) and fails to condense the myelin sheath^18,19^. Axons from young *LES* rats before postnatal 4 weeks are loosely covered by 2–3 thin layers of myelin, but then undergo progressive demyelination until, by three months of age, most axons are demyelinated^20^. Previous studies in the *LES* rat have shown that loss of tight and condensed myelination leads to alterations in ion channel expression at nerve terminals, and a reduction in conduction velocity and synaptic transmission in the central auditory circuit^8,9,21^. Here, we addressed that myelin deficiency in the cerebellum specifically reduced inhibitory synaptic transmission in the Purkinje cell-DCN synapse and consequently altered cerebellar output.

## Results

### Loss of MBP leads to dysmyelination and disrupts axonal structures of Purkinje cells

**Figure 1:**
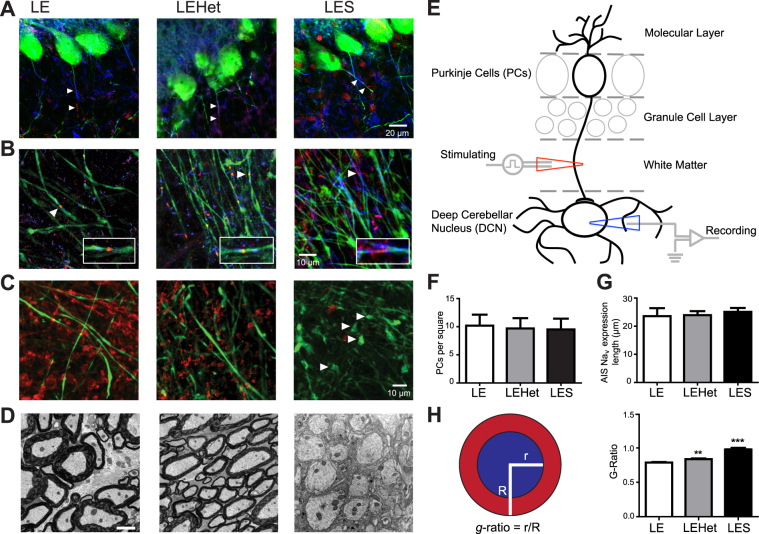
Structural changes of the Purkinje axon in the *LES* cerebellum (**A**). Calbindin (CB)-expressing Purkinje cells (green) in *LE*, *LEHet*, and *LES* cerebella. Caspr (blue) and Na_v_Pan (red) expression along Purkinje axons (arrowheads). (**B**) Purkinje axons expressing CB (green) in the white matter of the cerebellum of *LE*, *LEHet*, and *LES*. Arrowheads mark nodal and paranodal structures, expressing Caspr (blue) and Na_v_Pan (red), enlarged in inset. Inset width is 18 μm. While the nodal structure of Purkinje axons in *LE* and *LEHet* remain intact, Na_v_ and Caspr expression in *LES* Purkinje axons is diffuse. (**C**) CB-expressing Purkinje axons (green) and myelin basic protein (MBP) expression (red) in the white matter of the cerebellum of *LE*, *LEHet*, and *LES*. MBP expression is absent in the *LES* cerebellum, where spheroid swellings were observed in Purkinje axons (arrowheads). (**D**) EM images of myelination around Purkinje axons in *LE*, *LEHet*, and *LES*. Myelin was thinner in *LEHet* than *LE*, and nearly absent in *LES*. (**E**) Diagram of a Purkinje cell with dendrites that extend into the molecular layer and an axon projecting through the granule cell layer and white matter that synapses in the DCN. Recording electrode (blue) represents the recording site in Fig. 2 through 4. Stimulating electrode (red) indicates the stimulating site in Fig. 4. (**F**) Number of Purkinje cells per 304.5 μm × 304.5 μm square was not different between *LE*, *LEHet*, and *LES* animals. (**G**) There were no significant differences between the length of Na_v_Pan staining along Purkinje AIS between *LE*, *LEHet*, and *LES* animals. (**H**) Quantification of the *g*-ratio is calculated by dividing the radius of the axon (blue) by the total radius of the axon and myelin (red). The *g*-ratio quantified from electron microscopy (EM) images was significantly higher in *LEHet* and *LES* compared to *LE*.

Axons of Purkinje cells, located in the cortex of the cerebellum, follow white matter tracks and form synapses on DCN neurons (Fig. 1A,E). Immunohistochemistry and fluorescent imaging demonstrated how reduced MBP and myelin affected Purkinje cell number and axonal structures in *LE*, *LEHet*, and *LES* rats. We counted Purkinje cells in either cerebellar hemisphere of each genotype, in the same sagittal slices used for DCN experiments (see *Methods*). The number of Purkinje cells was not significantly different between genotypes (Fig. 1A,F), indicating the loss of MBP does not directly induce Purkinje cell loss. The expression of contactin-associated protein (Caspr)^22^ and Na_v_ channels in the proximal region near the soma of Purkinje cells, including the axon initial segment (AIS), was structurally not different among these genotypes (Fig. 1B). To quantify the AIS length, we measured the length of Na_v_ expression from three genotypes. There was no significant difference in the length (Fig. 1A,G). In *LES* Purkinje axons, nodal structure was disrupted, displaying dispersed Na_v_ channel expression at nodes (Fig. 1B). The major alteration in the integrity of axonal domains along myelinated fibers was observed in the pattern of Caspr expression, a prominent component of the paranodal axon glial junctions. *LE* and *LEHet* Purkinje axons, which were labeled with Calbindin, presented strong and well-defined labeling for Caspr at both edges of nodal Na_v_ channels, whereas Caspr labeling was weak and disperse around nodes of Ranvier from the *LES*. Thus it was difficult to detect the segregation of nodal and paranodal structures along *LES* Purkinje axons (Fig. 1B). In the *LES*, abnormal spheroid swellings along Purkinje axons were found in high quantity distal to the Purkinje soma, near the DCN, whereas few could be found more proximal to the soma (Fig. 1C). These swellings have been described as indication of axon deterioration leading to cerebellar damage and degeneration^23^. Thus, it could be speculated that the proximal axon close to the nerve terminal is more susceptible to myelin loss. In *LE* and *LEHet* rats, Purkinje axons were covered by myelin expressing MBP, whereas *LES* rats had no distinct MBP expressed along Purkinje axons (Fig. 1C). EM imaging of Purkinje axons revealed that the loss of MBP impacts myelination, as evidenced by the increased *g*-ratio in *LES*, and to a lesser extent *LEHet* (Fig. 1D,H). The ratio of the inner axonal radius to the total radius of the axon and myelin, *g*-ratio, is widely utilized as a functional and structural index of optimal axonal myelination. Thus, these structural deficits in Purkinje axons seen in the *LES* animals indicate that MBP-mediated compact myelination is critically important to maintain axonal health of Purkinje cells in the cerebellum.

### Lack of compact myelin increased spontaneous firing in DCN neurons

**Figure 2:**
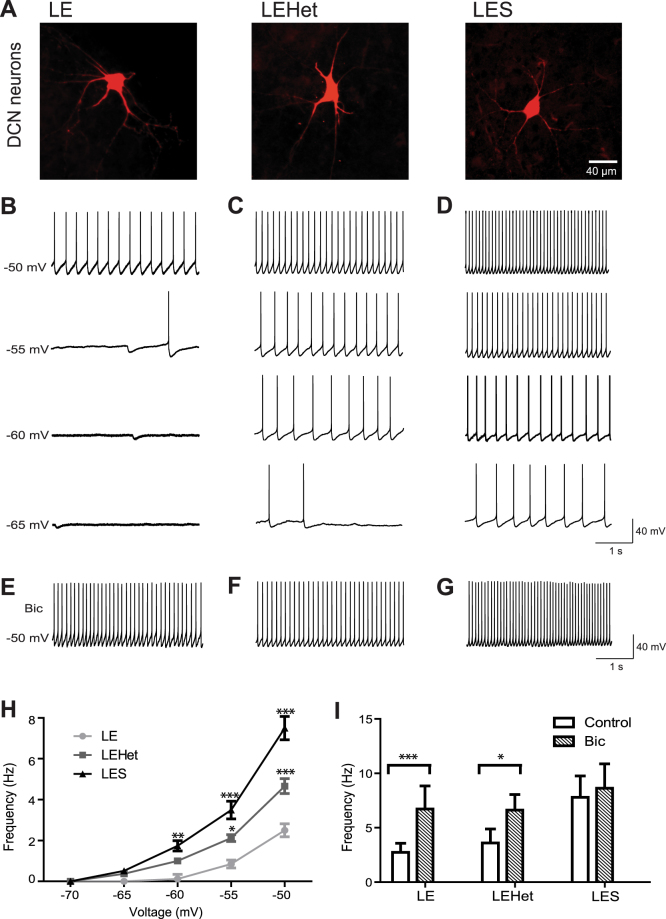
*LES* and *LEHet* DCN neurons display altered tonic inhibition and spontaneous AP firing. (**A**) Representative deep cerebellar nuclei (DCN) cells in *LE*, *LEHet*, and *LES*, which were filled with Alexa 568 during whole-cell recordings. (**B–D**) Whole-cell current clamp recordings of spontaneous APs at membrane potentials of −50, −55, −60, and −65 mV in DCN neurons of *LE* (**B**), *LEHet* (**C**), and *LES* (**D**) rats, demonstrating increased firing frequency in *LES*, and to a lesser extent, *LEHet* DCN neurons. (**E–G**) Spontaneous action potential firing in DCN neurons of *LE* (**E**), *LEHet* (**F**), and *LES* (**G**) rats at −50 mV in the presence of 10 μM bicuculline. (**H**) Summary of spontaneous firing frequency at various membrane potentials from −70 mV to −50 mV in *LE*, *LEHet*, and *LES* DCN cells. (**I**) The effect of bicuculline on spontaneous firing frequency (at −50 mV) in *LE*, *LEHet*, and *LES* DCN cells. *, **, and *** indicate p < 0.05, p < 0.01, and p < 0.001, respectively.

To examine the effect on cerebellar output resulting from alterations in Purkinje axon myelination due to MBP mutation, we recorded spontaneous AP firing from DCN cells of *LE*, *LEHet*, and *LES* rats and filled the cells with Alexa 568 (at P14–18, Fig. 2A). In the DCN, heterogeneous cell populations show various morphological and electrophysiological properties^13^. Based on their soma size and intrinsic properties, we chose larger DCN neurons for whole-cell recordings, which were defined by the diameter of soma (>25 μm), capacitance (>100 pF), and input resistance (241 ± 34.4 MΩ, n = 14).

In *LE* rat cerebellum, DCN cells fired spontaneous APs regularly at 2.5 ± 1.23 Hz (n = 9) at the physiological membrane potential of around −50 mV in *LE* rat brains (Fig. 2B). Spontaneous AP firing in *LE* DCN cells was not observed at membrane potentials below −60 mV in current-clamp recordings, but in 30% of recordings we detected inhibitory postsynaptic potentials (IPSPs) with amplitude of 5.2 ± 1.10 mV (n = 3). At −55 mV, spontaneous APs began to appear, and AP firing rate increased as the membrane potential depolarized to −50 mV, when no current was injected (Fig. 2B). *LEHet* and *LES* DCN cells fired spontaneous APs at membrane potentials as low as −65 mV, when hyperpolarizing current was injected, with increased firing frequency as the membrane potential depolarized (Fig. 2C,D). Average firing rate at −50 mV was 4.7 ± 1.33 Hz in *LEHet* DCN neurons and 7.5 ± 1.89 Hz in *LES* DCN cells (n = 7 *LEHet* cells and n = 9 *LES* cells, Fig. 2H). *LES* DCN cells displayed a higher firing rate at all membrane potentials compared with *LE* and *LEHet* (Fig. 2H). Furthermore, spontaneous IPSPs were not observable in *LEHet* and *LES* neurons. The results indicate that the extent of Purkinje axon myelination modulates spontaneous firing in DCN neurons, thus lack of myelination may impact the output of cerebellar signals from the DCN.

Spontaneous firing rate of DCN neurons is regulated by GABA-mediated tonic inhibition from presynaptic Purkinje cell terminals^24,25^. We tested whether increased spontaneous firing rate in *LEHet* and *LES* was due to changes in GABA_A_ receptor activation into these DCN cells from Purkinje cell terminals. The additional application of bicuculline, a GABA_A_ receptor antagonist, increased spontaneous firing rate from 2.7 ± 2.05 Hz to 6.7 ± 5.20 Hz (n = 6, t(5) = 2.756 p = 0.040, paired t-test, Fig. 2E,I) at membrane potential of −50 mV in *LE* DCN cells, indicating that tonic inhibitory input plays a role in spontaneous AP firing in normal DCN neurons. Firing rate in *LEHet* cells also increased from an average of 3.6 ± 3.14 Hz to an average of 6.6 ± 3.54 Hz in the presence of bicuculline (n = 6, t(5) = 3.244 p = 0.023, paired t-test, Fig. 2F,I), while firing rate in *LES* DCN cells did not significantly increase (n = 5, t(4) = 2.404 p = 0.074, paired t-test, Fig. 2G,I). The increased firing rate of both *LE* and *LEHet* DCN cells in the presence of bicuculline was not statistically different from the firing rate of *LES* DCN cells, suggesting that the GABAergic inhibitory input is the major factor underlying AP firing rate differences between genotypes. To avoid the non-specific effect of bicuculline, we tested the effect of another GABA_A_ receptor antagonist, SR 95531, on spontaneous firing in DCN cells. The effect of increased spontaneous firing in *LE* and *LEHet* cells was also observed in the presence of SR 95531, and an increase in firing rate was similar to that in the presence of bicuculline (data not shown). Together, these results indicated that the *LES* cerebellum lacking compact myelination had a reduction in GABA_A_ receptor activation in DCN cells, leading to hyper-excitability.

### The extent of myelin regulates GABA-mediated inhibitory synaptic transmission in DCN cells

**Figure 3:**
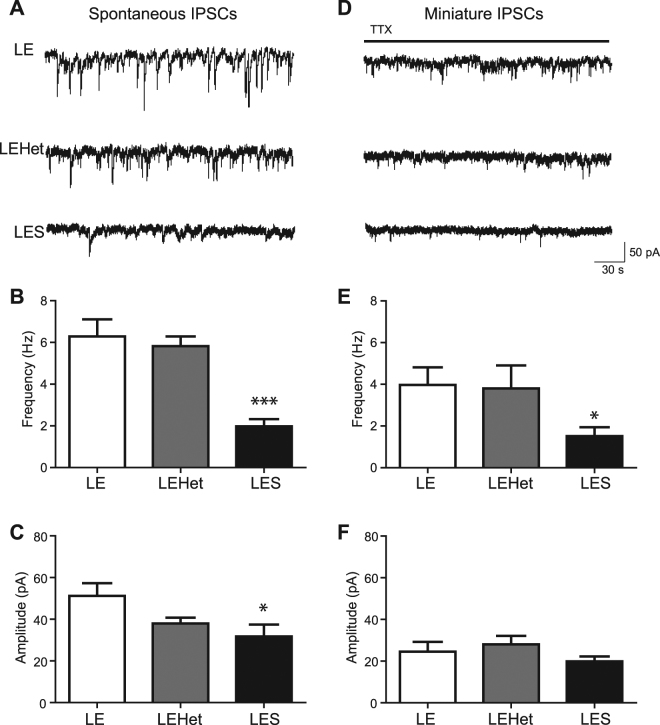
LES animals show reduced spontaneous inhibitory input into DCN cells. (**A**) Recordings of sIPSCs in DCN cells from *LE*, *LEHet*, and *LES* animals in the presence of CNQX (25 μM). (**B,C**) Summary of sIPSC frequency (**B**) and amplitude (**C**) in *LE*, *LEHet*, and *LES* DCN cells. (**D**) Recordings of mIPSCs in the presence of TTX (1 μM) in *LE*, *LEHet*, and *LES* DCN cells. (**E,F**) Summary of mIPSC frequency (**E**) and amplitude (**F**) in *LE*, *LEHet*, and *LES* DCN cells. *, **, and *** indicate p < 0.05, p < 0.01, and p < 0.001, respectively.

To determine whether the reduction in GABA_A_ receptor activation seen in *LES* DCN cells was due to reduced inhibitory input to these cells, we evaluated GABA-mediated inhibitory inputs from Purkinje cells by recording spontaneous inhibitory postsynaptic currents (sIPSCs) in DCN cells from *LE*, *LEHet*, and *LES* animals in the presence of CNQX. Average sIPSC frequency was 6.3 ± 0.82 Hz (n = 7) in *LE* and 5.8 ± 0.47 Hz (n = 7) in *LEHet* DCN neurons, while in *LES* DCN neurons sIPSC frequency was significantly less than *LE* at 2.0 ± 0.35 Hz (n = 7 per group, t(12) = 4.833, p = 0.0004, two-tailed t-test, Fig. 3A,B). This indicates that the *LES* DCN has fewer GABA-mediated inhibitory inputs. Average sIPSC amplitude was 51.3 ± 6.04 pA (n = 7) in *LE*, while in *LEHet* the amplitude of sIPSCs (38.0 ± 2.79 pA) was lower than, but not significantly different from, *LE* sIPSC amplitude (n = 7 per group, t(12) = 1.998, p = 0.0688, two-tailed t-test, Fig. 3A,C). *LES* sIPSC amplitude was significantly lower than *LE* at 31.8 ± 5.62 pA (n = 7 per group, t(12) = 2.362, p = 0.0359, two-tailed t-test, Fig. 3A,C). Average decay time of sIPSCs was similar among these groups (data not shown). The reduction in the amplitude and frequency of sIPSCs observed in *LES* DCN neurons indicate that proper myelination of the cerebellum regulates GABA-mediated synaptic inputs from Purkinje cell terminals to DCN neurons.

To test whether this reduction originates from reduced presynaptic release or reduced postsynaptic receptors, we recorded miniature IPSCs (mIPSCs) in the presence of TTX (1 μM) to exclude the effect of spontaneous APs. Average mIPSC frequency was 4.0 ± 0.85 Hz (n = 5) in *LE* and 3.8 ± 1.10 Hz (n = 4) in *LEHet*, while *LES* mIPSC frequency was significantly decreased (1.6 ± 0.43 Hz, n = 5, t(8) = 2.587, p = 0.0322, two-tailed t-test, Fig. 3D,E). The average amplitude of mIPSCs was not significantly different between *LES*, *LEHet*, and *LE* (n = 5 *LE* vs. 4 *LEHet* vs. 5 *LES*, Fig. 3D,F). This indicates that the number of inhibitory inputs or presynaptic GABA release is reduced in Purkinje terminal-DCN neuron synapses in the *LES* cerebellum, without alterations in the quantal size of vesicles or in postsynaptic receptor properties. Furthermore, in *LES* DCN cells, sIPSC and mIPSC frequency were not different. Thus, both sIPSCs and mIPSCs may be mediated by AP-independent vesicle release, indicating that few or no APs reach the *LES* Purkinje terminal due to failures spontaneous AP propagation.

### MBP mutation-induced dysmyelination reduced Purkinje cell AP efficacy

**Figure 4:**
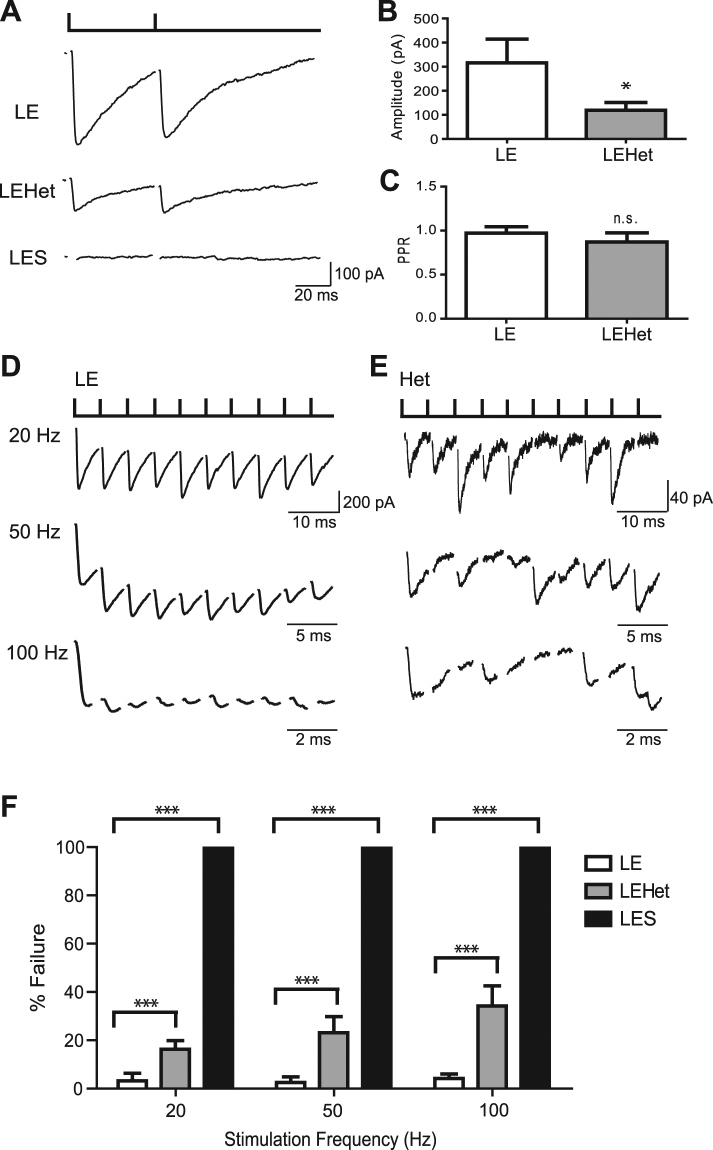
*LES* and *LEHet* show reduced inhibitory transmission at the Purkinje cell-DCN cell synapse. (**A**) Recordings of eIPSCs in DCN neurons evoked by stimulating Purkinje axon with bipolar stimulator placed in the cerebellar white matter in *LE*, *LEHet*, and *LES* rats. Paired pulse stimulation with an interval of 50 ms. Note no identifiable current in *LES* DCN cells in response to Purkinje axon stimulation. (**B**) Summary of eIPSC amplitude in *LE* and *LEHet* DCN neurons. (**C**) No significant difference between the paired pulse ratio (PPR) in eIPSCs from *LEHet* and *LE* DCN neurons. (**D**) and (**E**) Whole cell voltage clamp recordings of eIPSCs in DCN neurons of *LE* (**D**) and *LEHet* (**E**) rats after stimulation at 20, 50, and 100 Hz. (**F**) Summary of failure rate in *LE*, *LEHet*, and *LES* DCN cells at 20 Hz, 50 Hz, and 100 Hz. *LES* cells showed 100% failure rate. *, **, and *** indicate p < 0.05, p < 0.01, and p < 0.001, respectively.

To evaluate the MBP mutation-induced deficits in AP–mediated synaptic transmission in the Purkinje cell-DCN neuron synapse, we recorded evoked inhibitory postsynaptic currents (eIPSCs) in DCN cells by stimulating Purkinje axons in the white matter surrounding the DCN using a bipolar stimulator. Recordings were performed in the presence of AMPA/Kainate antagonist CNQX in order to isolate GABA-mediated inhibitory inputs from the Purkinje cells. In *LE* DCN neurons, eIPSCs had an average amplitude of 316.1 ± 98.4 pA (n = 6). The amplitude of eIPSCs gradually increased as the intensity of stimulus increased, thus we stimulated at the minimum intensity that evoked the maximum response. Compared with *LE*, *LEHet* DCN neurons had reduced eIPSC amplitude (119.5 ± 31.8 pA, n = 6 *LE* vs. 7 *LEHet*, t(10) = 1.902, p = 0.0268, two-tailed t-test, Fig. 4B). There was no significant difference in paired pulse ratio (Fig. 4A–C, *LEHet* average = 0.9 ± 0.3, *LE* average = 1.0 ± 0.1, n = 6 *LE* vs. n = 6 *LEHet*, t(10) = 0.8096, p = 0.4282, two-tailed t-test, Fig. 4C). Remarkably, *LES* DCN cells had no observable response to Purkinje axon stimulation (n = 9, Fig. 4A). To attempt to elicit a response from LES DCN cells, we increased stimulus intensity and stimulated closer to the DCN area, as close as ~70 μm from the recorded cell, to no avail. To determine whether conduction failure along the Purkinje axon causes this failure, we tested the extent to which AP propagation was impaired in *LES* Purkinje axons. LES Purkinje cells fired spontaneous APs, similarly observed in LE Purkinje cells. Recording the back-propagation action currents in the Purkinje cell revealed that APs can propagate along the axon within 200 μm of the soma in the *LES*, but failures occur at high frequency (50 Hz), while no failures occur in LE (Supplementary Figure 1). The severe phenotype of a lack of response from *LES* DCN cells to Purkinje axon stimulation is likely due to a combination of AP propagation and alterations in axonal and presynaptic properties.

Our previous study in the auditory brainstem showed that *LES* mutants have reduced AP conduction efficiency, which resulted in neurotransmission failures in a frequency-dependent manner^8,9^. To determine whether synaptic transmission failures in response to high frequency stimulation also occurred in Purkinje cell-DCN neuron synapses of *LEHet* animals, we stimulated Purkinje axons at 20, 50, and 100 Hz while recording from DCN cells in *LE* and *LEHet* rats. In *LE* DCN cells, the failure rate of eIPSCs was 3.2 ± 3.2% at 20 Hz, 2.6 ± 2.3% at 50 Hz, and 4.2 ± 1.9% at 100 Hz (Fig. 4D,F, n = 6). *LEHet* DCN cells had an average of 16.3 ± 3.6% failure at 20 Hz, 23.1 ± 6.6% failure at 50 Hz, and 34.2 ± 8.4% failure at 100 Hz (Fig. 4E, n = 7). The failure rate of eIPSCs was significantly increased at 20 Hz (n = 6 *LE* vs. 7 *LEHet*, t(11) = 2.686, p = 0.0212, two-tailed t-test), 50 Hz (n = 6 *LE* vs. n = 7 *LEHet*, t(11) = 2.737, p = 0.0193, two-tailed t-test), and 100 Hz (n = 6 *LE* vs. 7 *LEHet*, t(11) = 3.242, p = 0.0078, two-tailed t-test, Fig. 4D–F). This indicates that inhibitory synaptic transmission at the Purkinje cell-DCN neuron synapse is influenced by axon myelination in a frequency-dependent manner.

### Reduced inhibitory input to DCN neurons from Purkinje terminals in *LES* mutants

**Figure 5:**
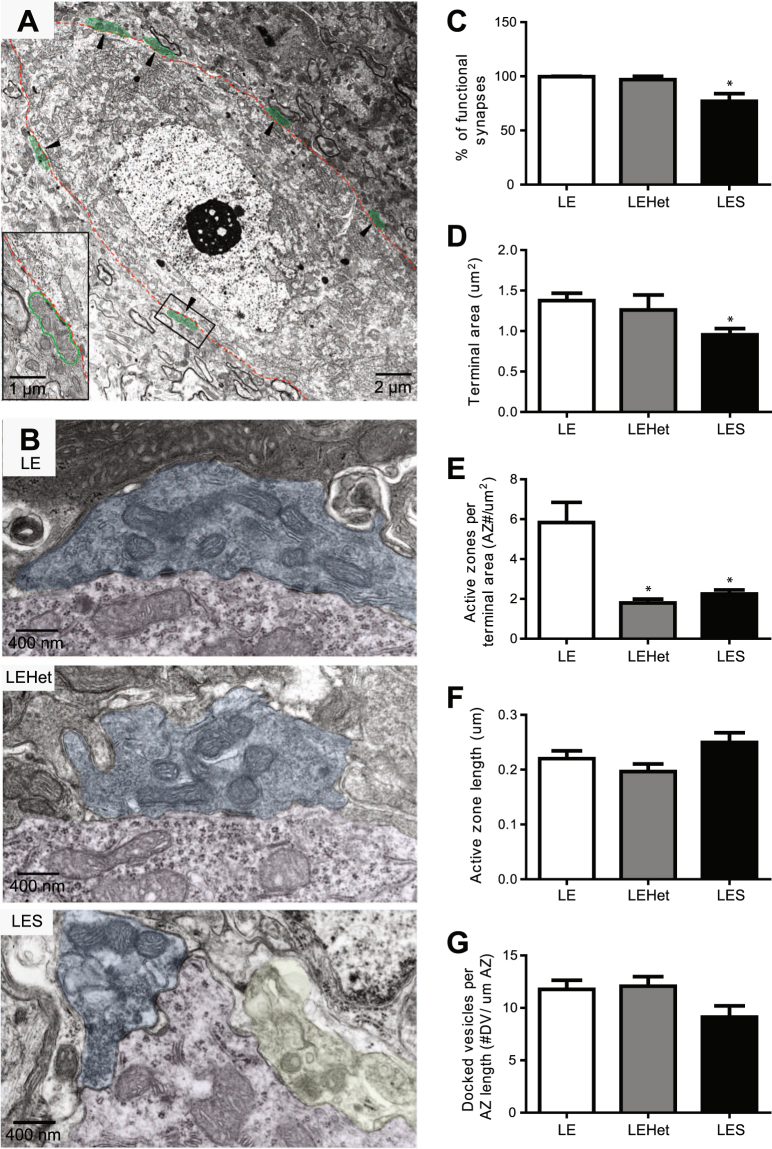
Purkinje cell-DCN neuron synapse structure disruptions in *LES* and *LEHet* animals. (**A**) EM image from the DCN of an *LE* rat. Arrowheads indicate examples of Purkinje cell terminals (colored green). (**B**) Electron microscopy images of Purkinje cell-DCN neuron synapses including all active zones in *LE*, *LEHet*, and *LES* rats. Yellow colored terminal indicates a degenerating terminal. (**C–G**) Summary of the number of functional (non-degenerating) Purkinje terminals per DCN cell (**C**), Purkinje terminal area (**D**), number of active zones per terminal (**E**), length of active zones (**F**) and number of docked vesicles (DV) per active zone (**G**) in *LE*, *LEHet* and *LES* rats. *, **, and *** indicate p < 0.05, p < 0.01, and p < 0.001, respectively.

The electrophysiological measurements in *LES* and *LEHet* mutants revealed reduced tonic inhibition and hyper-excitability of DCN neurons, which is associated with failure of spontaneous AP propagation along dys- or hypo-myelinated Purkinje axons. In addition to axonal deficits and conduction failures, a decrease in the number of functional Purkinje terminals or reduced GABA release at each Purkinje terminal can sufficiently contribute to synaptic transmission deficits at the Purkinje cell-DCN neuron synapse. To address the potential role of axon myelination in proper structural properties of nerve terminals and synaptic contacts, we performed ultrastructural analysis of the DCN area in *LE*, *LEHet*, and *LES* rats at P18, using electron microscopy. Purkinje cell terminals that form synapses on the DCN neuronal soma were recognized by their unique morphology (Fig. 5A), as previously described^26^. In the *LE* and *LEHet* animals, presynaptic terminals of Purkinje cells were filled with a dark filamentous matrix containing synaptic vesicles and mitochondria (Fig. 5B). However, in the *LES* DCN, Purkinje terminals displayed two distinct morphologies, which we characterized as “functional” terminals, with preserved ultrastructure containing mitochondria and synaptic vesicles similar to *LE*, or as “degenerating” terminals, with mitochondria showing abnormal morphology and lack of synaptic vesicles (Fig. 5B). Quantitative analysis of the total Purkinje terminal numbers around DCN cell bodies revealed no changes across genotypes, however in *LES* rats there was a significant 22% reduction of “functional” synapses compared to *LE* (n = 3 animals per group, t(4) = 3.247, p = 0.0315, two-tailed t-test, Fig. 5C) compared to *LE* controls. In addition, we found that axonal dysmyelination could affect the presynaptic apparatus at a single nerve terminal. Quantification analysis showed a significant reduction in the area of a single terminal in *LES* rats (n = 3 animals per group, t(4) = 3.515, p = 0.0246, two-tailed t-test, Fig. 5D), as well as the number of active zones per terminal in the *LES* rat compared to *LE* (n = 3 animals per group, t(4) = 3.466, p = 0.257, two-tailed t-test, Fig. 5E). Interestingly, the number of active zones was also altered in *LEHet* Purkinje terminals compared to *LE* (n = 3 animals per group, t(4) = 3.912, p = 0.0174, two-tailed t-test, Fig. 5E). However, the active zone length (n = 3 animals per group, F(2) = 2.999, p = 0.1251, one-way ANOVA, Fig. 5F) and the number of docked vesicles was not statistically different across genotypes (n = 3 animals per group, F(2) = 2.881, p = 0.1327, one-way ANOVA, Fig. 5G). Taken together, the ultrastructural analysis of the DCN demonstrated anatomically reduced inhibitory inputs to *LES* DCN neurons from Purkinje terminals, due to significantly increased population of degenerated synapses and decreased number of active zones in functional population of terminals that can potentially reduce presynaptic GABA release. Our results indicate that axonal myelin is crucial for the structural formation, maturation, or functional maintenance of Purkinje terminals synapsing onto DCN neurons.

## Discussion

In the *LES* rat, the absence of MBP prevents the multi-layered wrapping of the myelin sheath and resulted in either no myelin or a thin myelin sheath with a single layer. In addition to axonal deficits, lack of compact myelination reduced GABA release from Purkinje terminals, led to hyper-excitability of DCN neurons, and eventually induced an unstable output from the cerebellum in the *LES*.

The hyper-excitability of DCN neurons in *LES* animals, and to a lesser extent in *LEHet* animals, was influenced by a reduction of GABA_A_ receptor activation due to decreased inhibitory inputs of Purkinje terminals. Reduced frequency and amplitude of sIPSCs in *LES* DCN cells indicated that there were fewer inhibitory inputs into these cells or that there were significant alterations in GABA release from Purkinje cell terminals. The reduction in sIPSC frequency and amplitude is likely caused by failures of AP propagation along Purkinje axon in *LES*, also contributing to the lack of observable eIPSCs. In addition, our EM data provide an explanation for our electrophysiological findings regarding changes in functional properties. There was no difference in the total number of Purkinje terminals contacting DCN cells in *LE*, *LEHet*, and *LES* rats; however, there were a significant number of Purkinje terminals on *LES* DCN cells that were degenerated, lessening the number of functional inputs to these cells compared to the normal *LE* DCN neurons. This result suggests that axon myelination is able to modulate proper synapse formation and maintenance, and control synaptic inputs into the DCN in addition to AP propagation. Correspondingly, post-mortem assessment of the DCN of MS patients with marked demyelination showed anatomically the reduction in terminals contacting DCN cells, as well as degradation of synapses in the DCN^15^.

Changes in the amplitude of spontaneous postsynaptic currents could be associated with alterations in presynaptic properties including release probability, the quantal size of neurotransmitters, or the readily releasable pool size at presynaptic terminals^27,28^. Concerning a potential alteration in release probability, the paired pulse ratio between *LEHet* and *LE* was not significantly different, indicating that release probability is not responsible for the sIPSC amplitude reduction in the Purkinje-DCN cell synapse due to dysmyelination. To address potential alterations in the quantal size of neurotransmitter, the amplitude of mIPSCs was measured. mIPSCs are a result of single vesicle fusion, and their amplitude is an indicator of quantal size of vesicles containing neurotransmitter. mIPSC amplitude was similar among the three groups (*LE*, *LEHet*, and *LES*), indicating that quantal size in this synapse was not impacted by myelin deficiency. Changes in the vesicle number around the active zone, including the readily releasable pool size, can lead to reduced IPSC amplitude. However, EM imaging of the active zones in Purkinje terminals revealed that there were no differences in the number of docked vesicles in the Purkinje terminals of *LE*, *LEHet*, and *LES* animals. Interestingly, the terminal size and the number of active zones per Purkinje terminal were significantly decreased in *LES* mutants compared to those in *LE* animals. This alteration in structural properties of terminals may contribute to reduced GABA release from *LES* Purkinje terminals.

The reduced amplitude of eIPSCs in *LEHet* and no detectable eIPSCs in *LES* DCN cells is explained by conduction failure in Purkinje axons of *LEHet* and *LES* animals. In addition, high frequency stimulation of 100 Hz of Purkinje axons resulted in eIPSC failure of 34.2% in *LEHet* DCN cells, while nearly no failure (4.2%) was seen in normal *LE* DCN neurons, indicating that high frequency APs fail to propagate and invade the Purkinje terminal in *LEHet* DCN cells. We observed failures of back-propagated action currents in *LES* Purkinje axons, while there was no failure in *LE* Purkinje axons (Supplementary Figure 1). EM imaging revealed reduced myelin thickness in *LEHet* and *LES* Purkinje axons, an important contributor to conduction velocity^29,30^. Myelin shapes the structure of the node of Ranvier^31^, and thus demyelination leads to disrupted nodal ion channel distribution^32^. Ion channel expression and distribution along the axon is crucial for AP propagation and conduction velocity^31,33–35^. Previous studies in the MBP-deficient *LES* rat have revealed that ion channel distribution alterations and conduction failure at the nerve terminal critically resulted in synaptic transmission failure in the auditory brainstem^8,9,21^.

eIPSCs were not seen in *LES* DCN cells, despite the evidence from electrophysiological recordings of substantially detectable mIPSCs, as well as EM images displaying that Purkinje terminals contact *LES* DCN cells. It is likely that a contributor to this synaptic phenotype is a disruption of AP conduction along the deteriorating Purkinje axon. Immunofluorescence images of Purkinje axons in the *LES* cerebellum showed that the proximal region of Purkinje axons appeared predominately normal, despite a few spheroid swellings. However, toward the nerve terminal in the DCN area, the distal axons displayed abnormal structures of nodes and paranodes with a greater number of spheroid swellings, indicating severe Purkinje axon deterioration. This suggests that very few APs invade the Purkinje terminal in *LES* mutants, although APs can be generated at the AIS.

How is axonal dysmyelination associated with structural alterations in axons and presynaptic terminals? MBP deletion-induced dysmyelination causes deterioration of the Purkinje presynaptic terminal in *LES* animals, as well as structural alterations in this terminal in both *LES* and *LE* animals, through several potential mechanisms. Synaptic remodeling has been shown to occur as a result of synapse strengthening or weakening in the form of increased or decreased utilization of the synapse during development in the neuromuscular junction and CNS^36^. A recent study showed that TEA-induced long-term potentiation, which can be considered an increased utilization of the synapse, resulted in an increase in synaptic contacts and the number of active zones at mossy fiber boutons in the hippocampus^37^. Conversely, alterations in the Purkinje cell-DCN cell synaptic structure may occur due to increased AP failures at Purkinje terminals caused by axon demyelination. Lack of APs reaching Purkinje presynaptic terminals, and thus extreme underutilization of these terminals in *LES* animals could even lead to deterioration of the terminal itself. Activity-dependent synapse loss such as this has been previously described as a key mechanism in normal physiology, development, and pathology^36,38,39^. Alternatively, myelin-producing oligodendrocytes located near the axon or terminal may be important for formation, maintenance, or stabilization of the synapse. Several neurotrophic factors, such as BDNF, have impacts on synaptic maturation, including axon arborization and presynaptic differentiation^40,41^. Oligodendrocytes are one of the major providers of BDNF in the CNS^42–45^, and therefore abnormal maturation of oligodendrocytes due to MBP deletion may have a potential side effect on their roles in synapse maturation and stabilization.

The DCN is the sole output of the cerebellum. Neurons in this area take information from the cerebellar cortex and send signals to premotor areas, which affect motor function. Recent studies suggest that Purkinje cells relay highly synchronous signals, resulting in time-locked AP firing in the DCN^12^. Myelination is essential for this synchrony^6^. Abnormally increased AP firing from DCN neurons may result in infidelity of the signal and incoherent output from the DCN in the *LES*. In addition, synaptic transmission is disrupted as a result of presynaptic terminal structure alterations in the Purkinje cell-DCN neuron in both *LES* and *LEHet* animals. These findings suggest that myelination deficits alter axonal and presynaptic terminal structures, disrupt AP propagation, and obstruct functional neurotransmission.

## Materials and Methods

### Animals

Experiments using *Long-Evans Shaker* rats (*LES*, from Dr. Kwiecien, McMaster University, Ontario, Canada) of either sex between the ages of postnatal day 14 (P14) and P18 were approved and performed in accordance with the guidelines and protocols of the University of Texas Health Science Center at San Antonio (UTHSCSA) Institutional Animal Care and Use Committee (IACUC, approved protocol #14005×). Animals were maintained as heterozygotes, and crossed to generate pups homozygous for the MBP mutation. A fraction of offspring from the heterozygous animals exhibited the *LES* phenotype and could be easily identified by gross movement disorders (a distinguishable tremor) starting around 2 weeks following birth^19,46^. The shaking phenotype of the *LES* rat is autosomal recessive, present only in animals that are homozygous for the MBP mutation whereas wild type (*LE*,+/+) and heterozygous (*LEHet*,+/−) rats show no symptoms^18–20,47^.

### Slice preparation

Sagittal cerebellar slices (300 μm thick) were prepared from rat pups at P14–P18. After rapid decapitation, the cerebellum was removed from the skull and immersed in ice-cold artificial CSF (aCSF) containing the following (in mM): 125 NaCl, 2.5 KCl, 3 MgCl_2_, 0.1 CaCl_2_, 25 glucose, 25 NaHCO_3_, 1.25 NaH_2_PO_4_, 0.4 ascorbic acid, 3 myo-inositol, and 2 Na-pyruvate, pH 7.3–7.4, when bubbled with carbogen (95% O2, 5% CO2; osmolality of 310–320 mOsm/kg water). After cutting, the slices were transferred to an incubation chamber containing aCSF bubbled with carbogen and maintained at 35 °C for 30 min, and thereafter at room temperature.

### Electrophysiology

Whole-cell patch-clamp recordings were performed in normal aCSF at room temperature (22–24 °C) using the voltage or current-clamp mode of the EPC-10 (HEKA Electronik). Normal aCSF was the same as slicing aCSF, but with 1 mM MgCl_2_ and 2 mM CaCl_2_. For current clamp recordings, K-gluconate-based pipette solution contained the following (in mM): 130 K-gluconate, 20 KCl, 5 Na2-phosphocreatine, 10 HEPES, 4 Mg-ATP, 0.2 EGTA, and 0.3 GTP, pH = 7.3. For IPSC recordings, CsCl-based pipette solution included the following (in mM): 150 CsCl, 10 TEA-Cl, 0.2 EGTA, 1 MgCl_2_, 10 HEPES, 2 ATP, 0.3 GTP, and 10 phosphocreatine, pH = 7.3. Occasionally, pipette solution additionally contained an Alexa 568 (50 μM) to visualize the cell through dye-filling and subsequent imaging after whole-cell recordings. Patch electrodes had resistances of 3–4 MΩ, and the initial uncompensated series resistance (R_s_) was <25 MΩ. APs were elicited by a bipolar platinum/iridium electrode placed in the white matter fiber tract surrounding the DCN. Postsynaptic currents were measured under whole-cell voltage-clamp with a holding potential of −60 mV. For all experiments, R_s_ was <25 MΩ. Spontaneous postsynaptic currents were analyzed using Mini-analysis software (Synaptosoft Inc., Decatur, GA, USA; RRID: SCR_002184). Data were analyzed off-line and presented using Igor Pro (Wavemetrics, Lake Oswego, OR, USA; RRID: SCR_000325). Statistical significance was determined using either one-way ANOVA or Student’s t-test (GraphPad Prism; RRID: SCR_002798) and P values < 0.05 were considered significant. Data values are reported as means ± SEM. n represents the cell number.

### Immunohistochemistry

Cerebellar slices (100 μm thick) were prepared as for electrophysiology, but subsequently fixed with 8% (wt/vol) paraformaldehyde in phosphate buffer solution (PBS) for 20 min. Free-floating sections were blocked in 4% goat serum and 0.3% Triton X-100 in PBS for 1 h. For double immunohistochemistry, slices were incubated with antibodies for rabbit anti-calbindin (CB; 1:200; Cell Signaling Technologies, Cat # 13176), mouse anti-sodium channel (PanNav; 1:200; Sigma-Aldrich, Cat # S8809, RRID: AB_477552), guinea pig anti-Caspr (Caspr; 1:1,000)^22^, and mouse anti-MBP (MBP; 1:500; BioLegend, Cat # 808401, RRID: AB_2564741) overnight at 4 °C. Antibody labeling was reported by incubation with different Alexa dye–conjugated secondary antibodies (1:500, Invitrogen) for 2 h at room temperature. Slices were mounted onto Superfrost slides in photobleaching-protective medium (Fluoroshield; Sigma-Aldrich). Stained slices were viewed with laser lines at 488 nm (for green), 555 nm (for red) and 633 nm (for blue) using a 20×, 40×, or 64× oil-immersion objective on a confocal laser-scanning microscope (LSM-510, Zeiss). Stack images were acquired at a digital size of 1,024 × 1,024 pixels with optical section separation (z interval) of 0.5 μm and were later cropped to the relevant part of the field without changing the resolution.

### Purkinje Cell Count

Purkinje cell number was counted using 20×tiled images of entire sagittal cerebellar slices containing DCN from a cerebellar hemisphere. A 20 × 20 grid was constructed across the images, and 340 μm × 340 μm squares within the grid were randomly selected. CB-positive cells were counted only in the Purkinje layer, and only cells entirely in focus were counted. The number reported is the average of CB-positive cells per square in each genotype. Squares with zero CB-positive cells were not included. Blinded cell counting was performed in two to three slices (100 μm thick) from each animal (n = 3 in *LE*, n = 4 in *LEHet*, and n = 4 in *LES*).

### Transmission Electron Microscopy

Transmission electron microscopy was carried out as described in Saifetiarova *et al*.^48^. Briefly, animals were anesthetized and intracardially perfused with normal saline, followed by 5% glutaraldehyde/4% paraformaldehyde fixative. Brains were removed and post-fixed for another 2 weeks in the same fixative. 1 mm-thick samples of DCN area were dissected out and incubated overnight in 0.1 M sodium cacodylate buffer followed by incubation in 2% OsO_4_ solution and gradient ethanol dehydration. Finally, samples were incubated in propylene oxide, infiltrated with PolyBed resin and embedded in flat molds. After embedding, samples were submitted to the UTHSCSA Electron Microscopy Lab for further processing. Briefly, 90 nm sections were collected on copper mesh grids and poststained with uranyl acetate and Reynold’s lead. Samples were imaged on a JEOL 1230 electron microscope using Advanced Microscopy Techniques software. Elongated Purkinje cell terminals converging on cell bodies of DCN were recognized by presence of polymorphic synaptic vesicles in relatively dense matrix and collection of several profiles of slender mitochondria^26^. A total of 180–200 synapses were analyzed from 3 animals per group (*LE*, *LEHet*, and *LES* rats). The following parameters at final magnification of 30,000× were measured in each synapse: area of presynaptic terminal, number of active zones per terminal, active zone length, thickness of postsynaptic density and number of docked vesicles. Docked vesicles were defined as those within one vesicle diameter of the presynaptic active zone^49,50^. Only non-degenerated synaptic terminals were included in analysis of the *LES* group. The number of Purkinje cell terminals per neuron was quantified at magnification of 20,000×.

### Data availability

The authors declare that all data generated or analyzed in this study are available within the article. The data that support the findings of this study are available from the corresponding author upon reasonable request.

## Electronic supplementary material

Supplementary Figure
