# Supplementary material for: Myelination of Purkinje axons is critical for resilient synaptic transmission in the deep cerebellar nucleus
Source: Sci Rep. 2018 Jan 18;8:1022. doi: 10.1038/s41598-018-19314-0 (PMC5773691; doi:10.1038/s41598-018-19314-0)
Supplement: Supplementary file 1 — Supplementary Figure [file 41598_2018_19314_MOESM1_ESM.pdf]

Supplementary Figure

Myelination of Purkinje axons is critical for resilient synaptic transmission in the deep cerebellar nucleus

Tara Barron, Julia Saifetiarova, Manzoor A. Bhat and Jun Hee Kim*

Department of Cellular and Integrative Physiology, University of Texas Health Science Center, San Antonio, Texas 78229, USA

Running title: Myelination regulates synaptic transmission

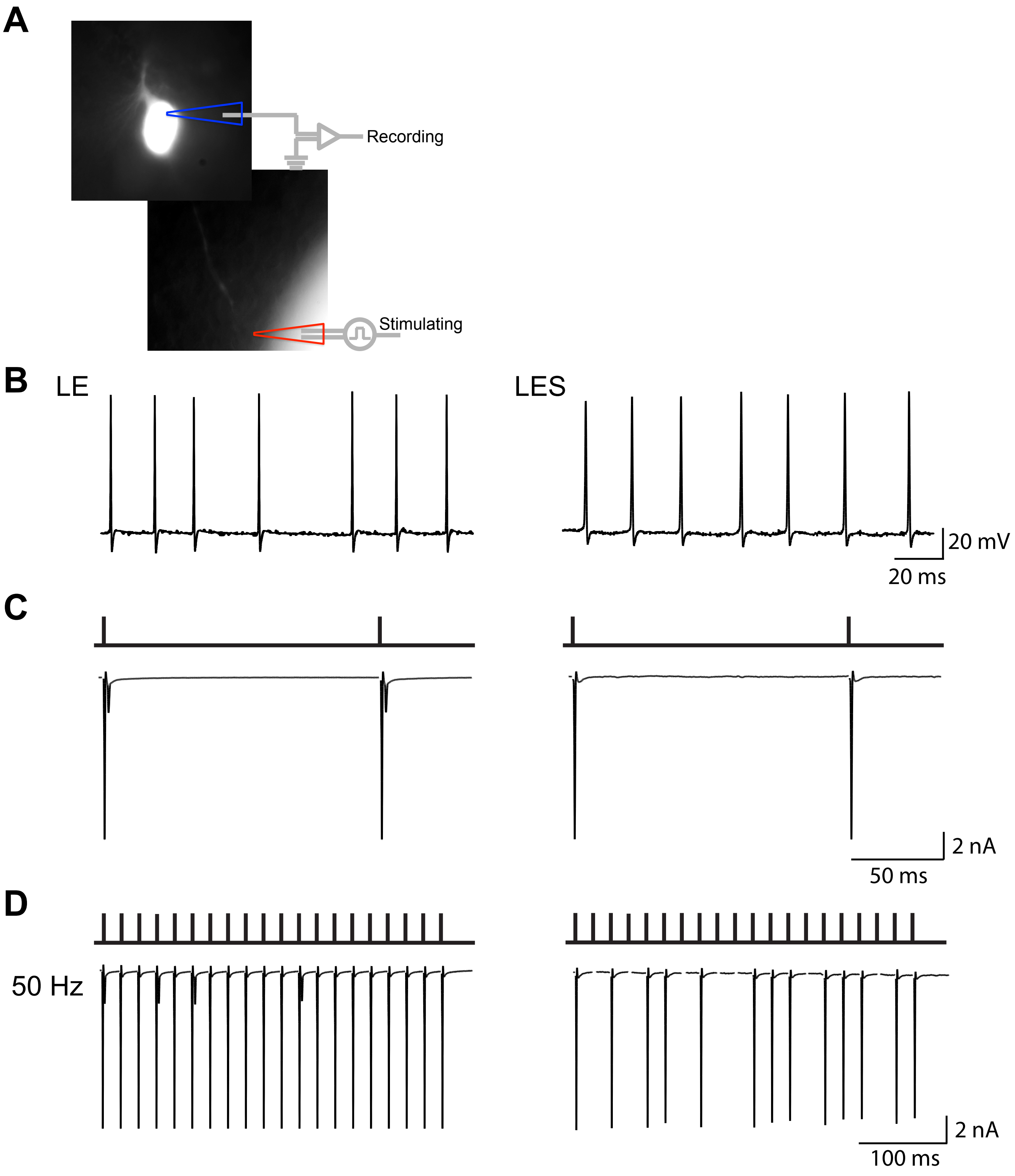

Supplementary Figure 1. AP firing and propagation in *LE* and *LES* Purkinje cells.
A. Purkinje cells were patched at the soma and filled with fluorescent dye to visualize the axon so that a stimulating electrode could be placed on the axon. **B.** Representative traces of spontaneous AP firing in *LE* and *LES*. **C.** Backpropagation action currents in Purkinje somata resulted when the axon was stimulated with a bipolar stimulating electrode in the presence of CNQX. **D.** Failure of backpropagation currents occurred in *LES* Purkinje cells when stimulated at 50 Hz, but did not occur in *LE* cells.
